# Exploring the Relationships Between Behavioural Biases and the Rational Behaviour of Australian Female Consumers

**DOI:** 10.3390/bs15010058

**Published:** 2025-01-10

**Authors:** Abhishek Sharma, Chandana Hewege, Chamila Perera

**Affiliations:** 1 Institute of Health and Management (IHM Australia), Melbourne, VIC 3051, Australia; 2 School of Business, Law & Entrepreneurship, Swinburne University of Technology, Melbourne, VIC 3122, Australia; chewege@swin.edu.au (C.H.); chamilaperera@swin.edu.au (C.P.)

**Keywords:** Australian female consumers, adaptive market hypothesis (AMH), rational decision-making, herding, overconfidence, Australian financial markets

## Abstract

The paper aims to examine the relationships between behavioural biases (such as overconfidence and herding) and the rational behaviour of Australian female consumers when making financial decisions. In doing so, the paper showcases the financial illiteracy of Australian female consumers when confronted with irregularities within the Australian financial markets. From a theoretical standpoint, the study adopts the notions of the adaptive market hypothesis (AMH) to understand the reasoning behind the relationships between behavioural biases (such as overconfidence and herding) and the rational behaviour of Australian female consumers when making decisions rationally. Using a quantitative approach, a structural equation modelling (SEM) was conducted on the proposed theoretical framework with a cleaned dataset of 357 Australian female consumers, which revealed that behavioural biases significantly influence each stage of rational decision-making when making financial decisions. More precisely, the structural equation modelling (SEM) showcases that herding behaviour has a significant positive relationship with the information search and evaluation of alternative stages when making financial decisions. However, overconfidence behaviour has a significant negative relationship with demand identification and evaluation of alternative stages when making financial decisions. Moreover, the findings also showcase that the proposed theoretical model closely fits with the data utilised, indicating that Australian female consumers do follow rational decision-making when making financial decisions. Additionally, the findings revealed that the education and income levels of Australian female consumers positively influence the stages of rational decision-making. The findings also contend that Australian female consumers have a risk-averse attitude (i.e., within three key hypothetical scenarios) towards financial decisions due to the presence of financial illiteracy. Hence, it is strongly suggested that financial institutions highlight the calculative benefits and returns from financial product purchases in advertising and promotions in a way that appeals to female consumer segments.

## 1. Introduction

Rationality occupies a central role within the fields of consumer behaviour and financial decision-making. In general, financial decision-making behaviour is viewed as a combination of a logical thought process and an individual’s rationality, which is supposed to provide them with the greatest utility and personal satisfaction (Dibb et al., 2021; Lučić et al., 2023; Mushinada, 2020; M. Sharma & Firoz, 2022). The initial definitions of rationality formulated were based on the rational choice theory, which suggests that individuals do often calculate the costs and benefits of any final decision (Homans, 1974; Lučić et al., 2023; Scott, 2000). According to Simon (1970), rationality empowers humans to make optimal decisions based on the available choices. However, several studies indicate that consumers are prone to face influences from behavioural biases such as herding and overconfidence in the presence of market instability within the financial markets (J. Kumar & Prince, 2023; Rubesam & Júnior, 2022). More precisely, the Australian financial markets are believed to be among the stable markets that rely upon the fundamental pillars of trust and transparency (Hayne, 2019; Keneley, 2020). Nonetheless, the investigations conducted by the Royal Banking Commission uncovered the prevalence of unethical practices within the Australian financial markets, with nearly 10,323 cases related to banking, superannuation and financial advisor sectors (ASIC, 2022; Hayne, 2019; Keneley, 2020; Sharma et al., 2022). In detail, nearly 50% of these cases reported (i.e., 5099 incidents) were related to consumer lending and personal finance sectors, and 11% of these cases reported (1139 incidents) were related to misconduct related to financial advice provided to consumers (ASIC, 2022; Hayne, 2019).

**Figure 1:**
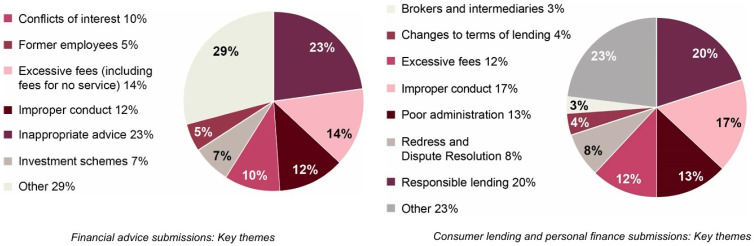
Financial misconduct reported to the Royal Banking Commission (source: (Hayne, 2019)).

Indeed, these reports also exposed various unethical practices and misconducts that are adopted by financial institutions and advisors when dealing with consumers (ASIC, 2022; Hayne, 2019) (See Figure 1). These unethical practices were mainly related to mis-selling of financial products along with inappropriate advice/disclosures, fraudulent activities (i.e., fees for services that are not provided, overcharged fees and underpaid interest on investments, misleading superannuation investments), which were primarily targeted towards consumers who have low levels of financial literacy in making decisions (ASIC, 2022; Hayne, 2019). Additionally, studies argue that the prevalence of unethical/manipulation practices within the financial industry creates a circumstance where an individual tends to mimic the behaviour (i.e., herding) of their peer members/friends or advice suggested by financial advisors (Ali-Rind et al., 2023; Chang & Lin, 2015; Galariotis et al., 2015). Furthermore, Basu and Dulleck (2020) also argued that market instabilities/crises, along with complex financial products, cause overconfidence among uninformed consumers when making financial decisions. Moreover, this misinformation/misattribution in financial markets tends to influence consumers with framing bias, in which they tend to underestimate the downside risks that are associated with financial investments (Ali-Rind et al., 2023; Basu & Dulleck, 2020). Similarly, studies argue that an increased level of information transparency from financial institutions (i.e., sharing information on product disclosure statements) is linked to positive affective evaluations among consumers when making financial decisions (Losada-Otálora & Alkire, 2019). In contrast, a lack of information transparency from financial institutions triggers a negative affective evaluation (i.e., anxiety) among consumers when making financial decisions (Lopez & Garza, 2023; Losada-Otálora & Alkire, 2019). For instance, a lack of transparency over financial products leads towards a generation of mistrust and an avoidance attitude towards financial decisions (Losada-Otálora & Alkire, 2019; Naveed et al., 2021). However, in several cases, consumers are often information overloaded or face contradictory/conflicting views (i.e., about a specific financial product) over social media platforms, which hinders an individual’s ability to make optimal financial decisions (Aggarwal et al., 2022; Frau et al., 2023; Sinnewe & Nicholson, 2023). Given this rapid change in the market sentiment and uncertainty caused within the Australian financial markets, it can be understood that manipulations from financial advisors have caused the prevalence of behavioural biases (i.e., herding and overconfidence) when making financial decisions (Hirdinis, 2021; S. Kumar & Goyal, 2016; Mushinada, 2020; Rubesam & Júnior, 2022). Moreover, these notions align with the propositions made by mainstream economists, who contend that making complex financial decisions within the higher market instability/volatilities can lead to the existence of herding and overconfidence behaviour among decision-makers. From a theoretical point of view of the prospect theory and adaptive market hypothesis (AMH), it can be confirmed that inefficiencies within the financial markets frequently influence an individual with a variety of behavioural biases, which are argued to deviate from an individual’s rational behaviour when making financial decisions (Lo, 2005, 2019; Mushinada, 2020). Additionally, several studies also state that uncertainty within financial markets is not the only reason behind the emergence of the irrational behaviour of decision-makers, but the presence of financial illiteracy influences an individual’s financial decision-making as well (Furrebøe & Nyhus, 2022; Goyal & Kumar, 2021; Lučić et al., 2023; Mireku et al., 2023).

Moreover, in comparison to men, women are reported to be facing several unique challenges when they deal with financial products, even if their financial situation is well-planned (Hira & Loibl, 2008; Lusardi & Mitchell, 2008; Sharma et al., 2023). Furthermore, one out of seven women tends to show higher impulsivity while making a financial decision. This is common among women who have lower levels of education and income (Lusardi & Mitchelli, 2007; Van Rooij et al., 2011). Additionally, it was found that women aged between 28 and 59 years of age have low levels of financial aspirations. They appear to be more interested in keeping an eye on their household and personal expenses (Hilgert et al., 2003; Loibl et al., 2007; Lusardi, 2008; Lusardi & Mitchell, 2008). Harrison (2016) also finds that Australian women who are aged between 18 and 24 and over 65 years working in blue-collar professions earning less than $20,000 per annum have very minimal levels of financial literacy. Overall, it is logical to assume that Australian women face several perceived complexities in dealing with financial products, as they have less financial control as compared to men, a greater discomfort when dealing with issues relating to loans and debts and less awareness of superannuation and other investments (Desigan et al., 2006; Hira & Mugenda, 2000; Kaur & Vohra, 2012; Mehta et al., 2001). Further, according to the Royal Commission reports, Australian women have been severely victimised by misconduct in the fields of insurance packages, personal loans and superannuation products (Hayne, 2019).

More specifically, studies do showcase that women are laggards within the financial decision-making domains, and they are often perceived to struggle to make effective financial decisions and are thus prone to behavioural biases (Furrebøe & Nyhus, 2022; Gudjonsson et al., 2022; A. Preston & Wright, 2023; West et al., 2023). While 30% of the worldwide population of women are considered to be financially literate, recent industry reports within Australia highlight that about twice the population (i.e., 34%) of Australian female consumers exhibit lower financial literacy levels as compared to men (Allianz, 2023; Vijay Kumar & Senthil Kumar, 2023). Moreover, higher levels of financial illiteracy are proven to escalate an individual’s herding and overconfidence behaviour, which influences an individual’s rational decision-making (Adil et al., 2021; Ateş et al., 2016; Lebdaoui et al., 2021). Numerous studies have stated that the prevalence of uncertainty and volatilities in financial markets not only restricts rational decision-makers from empowering their decisions but also allows the influx of behavioural biases, such as herding and overconfidence, to influence decision-making (Ghazani & Araghi, 2014; Mushinada, 2020; Urquhart & Hudson, 2013). Although some studies have indicated either a positive or negative effect on the potential impact of overconfidence and herding effect on investors’ decision-making, the relationship still remains unclear on what relationships do exist between behavioural biases (such as overconfidence and herding) and rational behaviour of Australian female consumers when making financial decisions (Niculaescu et al., 2023; Priem, 2021; Shah et al., 2021). Moreover, only a few studies appear to have shed light on the impact of behavioural biases on the rational behaviour of Australian female consumers when making financial decisions (de Oliveira Cardoso et al., 2023; J. Kumar & Prince, 2023). Therefore, it is of higher importance to understand the relationship between behavioural biases (such as overconfidence and herding) and the rational behaviour of Australian female consumers, who are primarily categorised as vulnerable groups. Based on this background, this study looks at the relationships that exist among the behavioural biases (such as overconfidence and herding) and rational behaviour of Australian female consumers when making financial decisions (i.e., in the presence of financial illiteracy). In doing so, the study depicts three hypothetical scenarios that reveal Australian female consumers’ risk perceptions and value assessments regarding financial investment decisions.

Based on the overview provided above, the paper is divided into seven sections. First, a brief introduction to the topic is provided, which articulates the research gap and the purpose for selecting this topic for this study. Next, a brief overview of financial vulnerability among Australian female consumers is presented, which is then followed by an overview of the role of rationality and behavioural biases on financial decision-making through the theoretical lenses of the adaptive market hypothesis (AMH). Later, the research methodology, sampling method and statistical techniques used in the current study to achieve the stated objectives are explained. Following that, a section of data analysis with findings is included, followed by a brief discussion of the findings generated and concluded with a summary of the key practical, academic and knowledge contributions.

## 2. Literature Review, Theoretical Background and Proposed Hypotheses

### 2.1. Financial Vulnerability Among Australian Female Consumers

Some studies related to gender stereotypes show that women are incapable of making optimal financial decisions (Carr & Steele, 2010; Daruvala, 2007; Grohmann et al., 2016). Moreover, recent reports highlight the persistent gender gap in financial literacy levels across the world, stating that men do better than women in financial decision-making (A. Preston & Wright, 2023; Worthington, 2013; Worthington & West, 2021). Moreover, with the growing complexities in financial products and uncertainties in financial markets, women still struggle in financial decision-making due to poor financial literacy and their restriction towards informal networks (Farrell et al., 2016). Although women have paved their way through the glass ceiling of economic discrimination through higher profile jobs in corporate sectors over the past decades, studies showcase that women perceive financial decision-making as a traumatic and stressful process due to their lower financial knowledge, fewer informal networks, lower self-efficacy levels and risk-averse behaviours (Furrebøe & Nyhus, 2022; Gudjonsson et al., 2022; Kaur & Vohra, 2012; Sotiropoulos & d’Astous, 2013).

Loibl et al. (2007) and Bettman et al. (1998) stated two strategies that women mostly adopt when making financial-product purchase decisions: emotion-focused coping and problem-focused coping. Emotion-focused coping describes an individual’s tendency to avoid further discussion regarding financial decisions (Kaur & Vohra, 2012; Loibl et al., 2007), and problem-focused coping is based on an individual’s desire to determine the best decision among all the alternatives (Kaur & Vohra, 2012; Loibl et al., 2007). The findings gathered from these studies conducted by Loibl et al. (2007) and Bettman et al. (1998) revealed that women who employ problem-focused coping tend to have a lexicographic decision style in which they prefer to make a judgement on the past performance of portfolios. However, in some cases, due to increases in the magnitude and frequency of negative emotions, women displayed strong avoidance behaviour, which greatly hindered their decision-making process. To these arguments, recent research also highlights that women are mostly conservative in nature when making financial decisions and prefer to invest in stable funds (Furrebøe & Nyhus, 2022; A. Preston et al., 2023; Yeh & Ling, 2022). As a result, it could be understood that women are largely vulnerable groups of the population who can be targeted and manipulated when making financial decisions.

Several theoretical perspectives have also been applied to understand women’s financial decision-making (See Table 1). These theoretical notions (i.e., theory of planned behaviour, transtheoretical model of change, health belief model, theory of reasoned action, risk-reduction model, role theory) when viewed with linkages to women’s financial decision-making display that women have a risk-averse attitude, a sense of fear and a lower level of confidence due to financial illiteracy when making financial decision-making (Kaur & Vohra, 2012; Loibl & Hira, 2006; Ozmete & Hira, 2011; Xiao, 2016). Further, Xiao et al. (2001) finds a linkage between the transtheoretical model of change and decision-making behaviour among women, proposing that women tend to have a risk-averse attitude towards financial decisions, due to which they hold on to losing stocks for longer and sell profitable stocks sooner. With numerous researchers finding theoretical linkages to decision-making styles among women, it can be summarised that most women tend to have a lower level of financial literacy, and a risk-averse attitude towards financial decision-making makes them vulnerable targets to behavioural biases and limits their ability to make decisions rationally (Dureha & Jain, 2022; Gupta & Goyal, 2024; Suresh, 2024).

Although this issue of financial illiteracy among women remains critical in developing countries, studies pertaining to OECD countries such as Australia showcase one of the largest gender gaps in terms of financial literacy among men and women (A. Preston et al., 2023; A. C. Preston & Wright, 2019; Worthington & West, 2021). Additionally, recent studies strike notable findings, which state that less than one in two (i.e., 48%) of Australian female consumers are financially literate as compared to two-thirds of the population (i.e., 63%) of men in Australia (A. Preston, 2020). Consequently, financial literacy acts as a hindrance, making women easily vulnerable targets to behavioural biases such as overconfidence and herding bias when making financial decisions. Therefore, it is worthwhile to investigate the relationships between behavioural biases (such as overconfidence and herding) and the rational behaviour of Australian female consumers when making decisions rationally.

### 2.2. Behavioural Biases and Adaptive Market Hypothesis

Understanding the influence of behavioural biases and estimating the efficiency in financial markets is critical in the presence of unexplained volatility in the financial markets. In this regard, Lo (2019) explained the linkages between rationality and the emergence of behavioural biases through the adaptive market hypothesis (AMH), which contends that individuals are rational actors who act/make decisions in their best interests but are quickly influenced by irrational factors due to market volatility and uncertainty (Lo, 2005, 2019; Mushinada, 2020). The theoretical notion implies that rational actors make mistakes when making financial decisions due to market uncertainties, but they also learn from their mistakes and adapt to new environments (Lo, 2005, 2019; Mushinada, 2020).

Moreover, studies also state that behavioural biases are visualised as a driving force that amplifies the inconsistencies within an individual decision-making behaviour. On theoretical grounds, Kahneman and Tversky (1979) state that individuals have distinct weights within their mindsets that are associated with gains and losses in financial decision-making. These allocations are made by investors based on an individual’s prior experiences, opinions, beliefs and information (Litimi, 2017; Nofsinger, 2005; Nofsinger & Sias, 1999). Further, critical domains of research on behavioural and psychological influences showcase critiques on cognitive dissonance (Festinger, 1957), self-perception (Nisbett & Wilson, 1977), behavioural biases (S. Kumar & Goyal, 2016; Zahera & Bansal, 2018), emotional misattribution (Andrade & Ariely, 2009; Lerner et al., 2015) and appraisal dimensions (DeSteno et al., 2000; S. Han et al., 2007; Lerner et al., 2004), which influence financial decision-making. While all these dimensions do provide a shared understanding of the irrational behaviour of a rational individual when making financial decisions, the current study focuses on understanding the relationships that exist among the behavioural biases (such as overconfidence and herding) and rational behaviour of Australian female consumers when making financial decisions (i.e., in the presence of financial illiteracy).

### 2.3. Rationality and Financial Decision-Making

Within behavioural finance, individuals are usually viewed as rational agents who follow specific procedures before reaching financial decision-making (Cushman, 2020; S. Kumar et al., 2022; Lučić et al., 2023; Mushinada, 2020). This rational behaviour of an individual has been argued to be the consequence of systematic decisions based on certain logic and probabilistic estimations. Previous researchers within the domains of decision-making denote that the rational decision-making process involves three major steps (i.e., demand identification, information search, and alternative evaluation), which an individual is argued to follow before making a final decision (S. Kumar & Goyal, 2016; Lin, 2011; Mintzberg et al., 1976; Mushinada, 2020). In detail, a rational decision-maker starts to recognise the nature of the problem (i.e., demand identification), which is followed by seeking more relevant information (i.e., information search) to make optimal financial decisions (S. Kumar & Goyal, 2016; Lin, 2011; Mushinada, 2020; M. Sharma & Firoz, 2022). Additionally, effective information search not only allows an individual to have several options to make a choice but also allows them to make proper risk and value assessments (i.e., evaluation of alternatives) before reaching an optimal choice (Rezabakhsh et al., 2006).

Traditionally, an individual’s purchase behaviour in financial-product markets has been associated with consumer inertia or passivity (Pousttchi & Dehnert, 2018; Solomon et al., 2012). In general, the need (i.e., demand identification) for having a financial product purchase or making a financial decision is driven by the consumer’s need, lifestyle choices, risk preferences and financial aspirations (Beckett et al., 2000; Devlin, 1998; Howcroft et al., 2003). From a broader perspective, financial decisions related to insurance purchases (i.e., life insurance, health insurance, home insurance, vehicle insurance) are generally linked with a perspective of financial security and minimising risks in uncertain situations (Chui & Kwok, 2008; Driver et al., 2018). However, certain financial products such as loans and mortgages (i.e., car loans, housing mortgages) are preferred by consumers to acquire an asset and to ensure long-term stability (Chui & Kwok, 2008; Driver et al., 2018). From an investment perspective, consumers and their demand identification related to industry funds (i.e., superannuation funds) and retail funds (i.e., private investment funds) are linked to higher returns and profit maximization (Gallery et al., 2011). In detail, retail funds are often preferred by consumers for higher returns, but they are often associated with higher market volatility (Cummings, 2016). Meanwhile, industry funds are usually linked to stable returns but have lower returns due to greater stability in the financial markets. Moreover, due to the increasing complexity and manipulation adopted in financial markets, the rational decision-making process of an individual is often influenced by over-reliance on financial advisors/experts/peer members, which limits them from making optimal decisions (Hayne, 2019; O’Brien, 2019).

Viewing consumers as rational agents suggests that they can make better decisions when provided with a maximum number of options (McShane & Sabadoz, 2015; Wright et al., 2006). For example, consumers can better distinguish which product to buy when there are around 20 options rather than just two. Having more choices allows a consumer to use their rationality best (Kucuk & Krishnamurthy, 2007; McShane & Sabadoz, 2015). However, studies do state that decision-makers often lack the ability to get reliable sources of information that can allow them to make effective financial decisions. Additionally, some researchers argue that restricted market transparency does not allow an individual to exercise their decision-making powers rationally (Brunnermeier et al., 2009; Juurikkala, 2012). Though social media platforms and engagement groups are useful in providing information regarding certain purchases (Kucuk & Krishnamurthy, 2007; Rezabakhsh et al., 2006; Suki & Suki, 2017). Information about financial products is very limited, and consumers cannot access the positive/negative experience of a certain financial product (Byrne, 2005; Devenow & Welch, 1996). As a result, based on the above literature and several studies, it can be concluded that each stage of rational decision-making not only allows a decision-maker to have proper risk and value assessment but also allows them to gain empowerment while making financial decisions (S. Kumar & Goyal, 2016; Lin, 2011; Mushinada, 2020). Hence, the following hypotheses are proposed:

**H1:**  *Demand identification within Australian female consumers has a significant relationship with their information search when making financial decisions.*

**H2:**  *Information search among Australian female consumers is significantly related to their evaluation of alternatives when making financial decisions.*

### 2.4. Rational Decision-Making and Behavioural Biases

In general, behavioural finance depicts the linkages of behavioural and psychological aspects and their influence on financial decision-making. Previous research conducted within the field of financial decision-making does not show complete agreement on the rational behaviour of consumers. Some research argues that the rational behaviour of an individual is not free; however, it is always resolute. From a psychological point of view, the consumer decision-making process can also be simultaneously rational and irrational (Allen, 2002; Labrecque et al., 2013; Lučić et al., 2023; Mushinada, 2020; Riquelme, 2001). On these lines of discussion, normative theorists have argued that a rational decision-maker utilises probabilistic estimation (Kahneman et al., 1982) and logical reasoning (Tversky & Kahneman, 1989) to make decisions. However, Simon (1955) and Kahneman and Tversky (1979) bridged ideas on how bounded rationality and behavioural biases, such as heuristics and disposition effects, play a significant role in financial decision-making (S. Kumar & Goyal, 2016; Sahi et al., 2013; Zahera & Bansal, 2018). Several researchers have debated that human actions are purely rational, but the notion has been criticised by psychological theorists who argue that “bounded rationality” is often used as a fashionable term for what are actually human irrationalities (Gigerenzer & Todd, 1999; Kahneman, 2003). Furthermore, studies do contend the fact that the increase in volatility and uncertainty can influence an individual’s decision-making with several behavioural biases (i.e., herding and overconfidence) when making financial decisions (Jan et al., 2022).

#### 2.4.1. Rational Decision-Making and Overconfidence Bias

In general, overconfidence bias is linked with situations in which the decision-makers tend to be over-optimistic about the possibilities and outcomes that are associated with a financial decision (Baker et al., 2019; Lin, 2011). A considerable number of studies have stated that having improper financial knowledge or ignoring publicly available information leads to the generation of overconfidence among decision-makers. Overconfidence bias is seen to influence each stage of rational decision-making. Primarily, Mushinada (2020) asserts that individuals tend to overestimate returns (i.e., during the demand identification stage) for financial investments, which in-turn allows them to invest in financial products that are associated with unexpected returns. Moreover, Grežo (2021) suggests that overconfidence is often associated with over-precision, over placement and overestimation. Additionally, overconfident decision-makers tend to incline towards private information (i.e., during information search stages) that is gathered from internal sources rather than relying on public information. Lastly, S. Kumar and Goyal (2016) stated that overconfident decision-makers tend to underweight/overweight returns during the evaluation stages that are associated with financial investments (Inghelbrecht & Tedde, 2024). Moreover, Inghelbrecht and Tedde (2024) state that overconfident individuals with lower levels of financial literacy tend to remain restricted in exercising their decision-making powers and making well-informed decisions. Moreover, based on the background provided above, it can be assumed that overconfidence bias, along with the presence of financial illiteracy among Australian female consumers, can restrict them from making risk and value assessments in financial decision-making (Baker et al., 2019; S. Kumar & Goyal, 2016). Hence, the following hypotheses are proposed:

**H3:**  *Demand identification within Australian female consumers has a significant relation to overconfidence bias when making financial decisions.*

**H5:**  *Information search among Australian female consumers has a significant relation to overconfidence bias when making financial decisions.*

**H7:**  *Evaluation of alternatives within Australian female consumers has a significant relation to overconfidence bias when making financial decisions.*

#### 2.4.2. Rational Decision-Making and Herding Bias

Herding refers to the tendency of decision-makers to imitate the decisions of peer members or other investors (Baker et al., 2019; S. Kumar & Goyal, 2016). Several studies have also shown that uncertain financial markets and volatility, as well as inadequate knowledge of financial products, influence individuals to mimic the investments (i.e., demand identification stage) of peer members. While financial literacy is a key factor in limiting women’s ability to fully exercise their decision-making powers, limited information about complex financial investments (i.e., information search stage) makes them vulnerable to biases such as herding when making decisions (Baker et al., 2019; Hsu et al., 2021). Some studies also suggest that men appear to have more overconfidence bias than women investors, whereas women appear to have a stronger herding bias when making financial decisions than men (Baker et al., 2019; Barber & Odean, 2001; S. Kumar & Goyal, 2016; Lin, 2011). Moreover, Rubesam and Júnior (2022) state that market instability and volatility cause individuals to neglect their own information and imitate the behaviours of peer members/investors during financial decision-making. As a result, based on the background of the study, it can be believed that Australian female consumers tend to display herding behaviour and struggle to exercise their rationality when making financial decisions. Hence, the following hypotheses are proposed:

**H4:**  *Demand identification within Australian female consumers has a significant relation to herding bias when making financial decisions.*

**H6:**  *Information search among Australian female consumers has a significant relation to herding bias when making financial decisions.*

**H8:**  *Evaluation of alternatives within Australian female consumers has a significant relation to herding bias when making financial decisions.*

**Figure 2:**
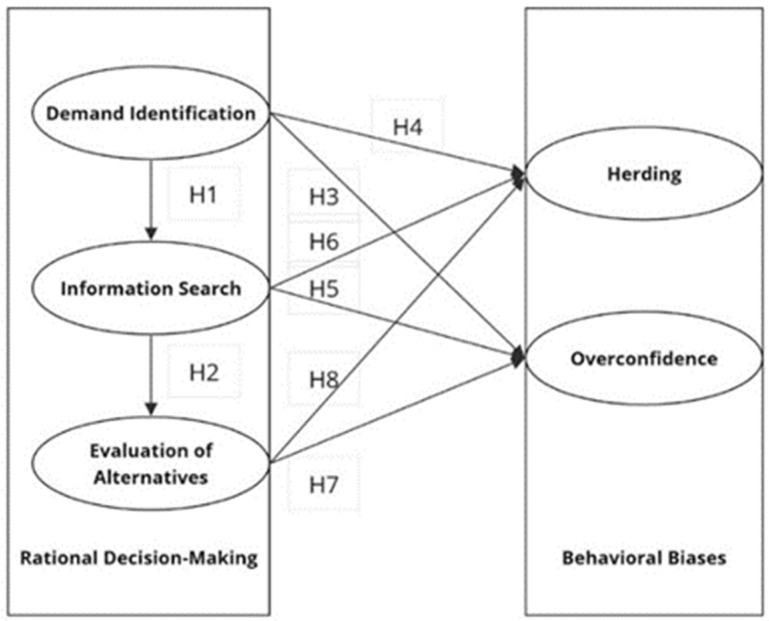
Conceptual framework.

More precisely, the conceptual framework (see Figure 2 below) is built on the theoretical notions of the adaptive market hypothesis (AMH), which seeks to uncover the relationships between behavioural biases (i.e., herding and overconfidence) and rational decision-making stages when making financial decisions. As discussed above, rational decision-making is comprised of three stages/constructs (i.e., demand identification, information search and evaluation of alternatives) and is argued to be influenced by behavioural biases (i.e., herding and overconfidence) when making financial decisions. It postulates that the relationships between behavioural biases (such as overconfidence and herding) and financial illiteracy influence the rational behaviour of Australian female consumers when making financial decisions.

## 3. Methodology

### 3.1. Data Collection and Survey Administration

The survey is designed to capture responses from Australian female consumers about their decision-making styles when making financial decisions. According to previous studies, women aged between 28 and 59 years have low levels of financial aspirations (Allianz, 2023; Loibl et al., 2007; Lusardi & Mitchell, 2008). In Australia, women who are aged between 18 and 24 years are reported to have very minimal levels of financial literacy and are vulnerable to misconduct in the financial market (Harrison, 2016; Hilgert et al., 2003; Loibl et al., 2007; Lusardi & Mitchell, 2008; A. Preston & Wright, 2023). As a result, Australian female consumers who are above the age of 18 years are considered suitable for this study. After seeking the final ethics approval, the survey link was made accessible to the original population. The developed survey link was then circulated through e-mails and social media platforms such as Facebook and LinkedIn. Additionally, the web link was also provided to Qualtrics to circulate the survey among the targeted population (i.e., Australian female consumers 18 years or above) within Australia.

### 3.2. Data Screening

In total, the online survey in the current study had 434 respondents. However, before the commencement of any data analysis, it is of utmost importance to use sophisticated techniques to clean the dataset (Hair et al., 2010; Leys et al., 2018). To resolve these issues, a four-step approach of data screening was performed in this study so that structural relationships could be estimated on a refined dataset. To begin with, 46 responses were removed from the current dataset due to incomplete responses (i.e., more than 60%) and lower reliabilities in this study. Secondly, 13 responses were found to be associated with disengaged responses, which were removed from the current dataset. Thirdly, it is argued that outliers can be categorised as either being univariate or multivariate variables (Joseph et al., 2010). For univariate and multivariate variables, conventional criteria of standardising the variables were considered, and then z-scores >3.29 or <−3.29 were removed from the dataset as they were extreme outliers (Osborne & Overbay, 2004). Fourthly, the Mahalanobis distance approach was performed on the sample set, and samples with sig < 0.001 were considered outliers (De Maesschalck et al., 2000; Leys et al., 2018). Hence, in total, 18 responses were found as outliers and were removed from the study. Finally, using SPSS, tests for normality, linearity and multicollinearity were performed on the current dataset, and no such issues were found. After the implementation of the above data cleaning techniques, a total of 357 responses were considered as refined datasets, which were utilised for the analysis during the current study.

### 3.3. Sampling

A non-probability sampling technique is utilised to provide wider access to Australian female consumers and tackle the burden of reduced responses, as participants may feel uncomfortable in disclosing their financial literacy levels and their decision-making styles when purchasing financial products (Lehdonvirta et al., 2021; Van Selm & Jankowski, 2006). Additionally, the non-probability sampling technique not only allows the study to gather rich responses but also ensures that the data gathered is cost-effective.

Alternatively, this sample size could be justified by using the following norm as well:

Female population in Australia (18–59 years) (ABS, 2022) = 7,496,086

*N* = 7,496,086, Z = 1.96 (95% confidence), *p* = 0.5, confidence level = 95%, margin of error (e^2^) = 5%

$$n_{0}=\frac{Z^{2}\times p\times (1-p)}{e^{2}}\;N=\frac{n_{0}}{1+\frac{n_{0}}{N}}$$

**Sample Size Determination Using Finite Population Correction Factor**

Approx. sample size = 384

As structural equation modelling (SEM) is utilised as the preferred method for statistical analysis, it can be understood that a minimum sample size of 300 or above is desirable to achieve the relationship within the measurement model. On these notions, Hair et al. (2010) also state that structural models with seven or fewer constructs should have a minimum sample size of 300 respondents to derive acceptable statistical outcomes. Lastly, previous studies have undertaken sample sizes ranging from 260 to 386 valid responses to investigate the interactions between rational decision-making and demographic variables when making risky financial decisions (S. Kumar & Goyal, 2016; Lakshmi et al., 2013; Melović et al., 2022). As a result, based on the above justifications, it could be determined that the sample size chosen within this study is appropriate to investigate the relationship between rational decision-making and behavioural biases when making financial decisions.

### 3.4. Adapting SEM Techniques for Data Analysis

According to Leedy et al. (2014) and Bryman (2016), quantitative research is the dominant type of methodology in the social sciences. Within sophisticated quantitative techniques, SEM is widely used to analyse multivariate datasets (Joseph et al., 2010). Typically, SEM employs two models: a structural model, which shows the relationships between the various latent constructs and a measurement model, which shows the relation between the latent constructs and their measures (Joseph et al., 2010). Before running SEM models, a factor structure should be constructed in a measurement model based on exploratory and confirmatory factor analysis (Gefen et al., 2000). Exploratory factor analysis (EFA) is mostly used to develop scales, which are validated with the help of confirmatory factor analysis (CFA). Additionally, model-fit measures such as RMSEA, AGFI, GFI and CFI help the researcher construct a robust model (Gefen et al., 2000; Joseph et al., 2010). Moreover, only a few studies have looked at the impact of behavioural biases (such as herding and overconfidence) on the rational decision-making process of Australian female consumers when making financial decisions. As a result, SEM fits as the best statistical technique that can be utilised for statistical validation and employed for deducing the relationship of the proposed constructs in the conceptual framework with the decision-making powers of Australian women when making financial-product purchase decisions.

### 3.5. Development of the Survey Instrument

The survey questionnaire implemented in this study is developed using validated scales from previous studies to investigate the relationships between behavioural biases (such as overconfidence and herding) and the rational behaviour of Australian female consumers when making decisions rationally. The survey is segregated into three different parts. The first segment of the survey involved questions on three hypothetical scenarios that were adapted from Gambetti and Giusberti (2012) to investigate the risk and value assessments of Australian female consumers when making financial investments. Next, the second segment of the survey is adapted from Lin (2011), and it includes questions about the stages of rational decision-making (i.e., IDENT-3 items, INFO-2 items, EVAL-3 items) when making financial decisions. Additionally, the composite reliabilities of each of the sub-scales adapted within this survey ranged from 0.62 or above, which ensures that the latent variables utilised in this study are reliable (Bryman, 2016; Hair et al., 2010). Lastly, the final segment of the survey included questions from financial literacy questions that were adapted from Van Rooij et al. (2011) to measure the basic literacy levels (i.e., numeracy, interest rates, inflation, compounding interest, and the time value of money) of Australian female consumers. The survey was then circulated across FB, LinkedIn, and other social media platforms. Further, to have access to a wider audience, Qualtrics was chosen to circulate these surveys to the designated population (i.e., Australian female consumers) and have their views on their rational behaviour when making financial decisions.

## 4. Findings and Discussion

The current study revealed the relationships between behavioural biases (such as overconfidence and herding) and the rational decision-making process of Australian female consumers when making financial decisions (i.e., in the presence of financial illiteracy). From the demographic profiles of respondents, it can be stated that 8.6% (n = 31) of Australian female consumers were under 25 years of age, followed by 26% (n = 93) of respondents who were from the age bracket of 26–35 years of age. Additionally, 22.4% (n = 80) were from 36 to 45 years of age, followed by 28.2% (n = 101) and 14.5% (n = 52) respondents who were within the age brackets of 46–65 years of age and 65 years or above, respectively. Moreover, from an income level perspective, it can be stated that 28% (i.e., 100) of Australian female consumers had a low income level (i.e., less than 20,000$), which is followed by 59.10% (i.e., 211) of Australian female consumers who had a middle income level (i.e., 20,000$–75,000$) and 12.88% (i.e., 46) of Australian female consumers who had an upper income level within Australia. Additionally, it can be inferred that 0.8% (i.e., 3) of Australian female consumers had an educational level up to schooling, which is followed by 56.3% (i.e., 201) of Australian female consumers who had an education level up to a certificate (O/A levels), which was followed by 31.6% (i.e., 113) and 11.2% (i.e., 40) of Australian female consumers who had undergraduate or postgraduate levels of study, respectively. Additionally, based on the above-mentioned statistics on Australian female consumers, the upcoming sub-sections detail the three key hypothetical scenarios that measure the risk perceptions and forecasting trends of Australian female consumers when making financial decisions.

### 4.1. Responses to Hypothetical Scenarios and Financial Literacy Levels Among Australian Female Consumers

With the increasing number of studies on the influence of behavioural biases on financial decision-making, it is important to understand the risk perceptions and value assessments of women when making financial decisions (Priem, 2021; Riquelme, 2001; M. Sharma & Firoz, 2022). Within the theoretical grounds of rationality and decision-making, it is widely argued that risk and value assessments are done by an individual to gain the expected utility in a financial decision. However, as stated in an earlier discussion on the volatilities and uncertainties in financial markets, individuals face behavioural biases when making financial decisions. As a result, the participants were given three hypothetical scenarios in which they responded to investment trends and predictability to assess the risk perceptions and value assessments of Australian female consumers (See Table 2).

Based on the findings from Table 2 above, it can be stated that most respondents (i.e., 98%) in the first hypothetical scenario exhibited a conservative attitude towards financial investments. However, the responses to the second and third hypothetical scenarios highlight that even situations of immediate risk, such as gain/loss, cause respondents to hold on to their investments for a few days to a week rather than sell them immediately. Lastly, with the extreme volatility and uncertainties in the financial markets, responses from Australian female consumers confirm that it is very difficult to forecast investment trends and their associated returns.

Additionally, to fully comprehend the financial literacy levels, questions related to the basic financial literacy concepts such as interest rates, inflation, compounding interest and the time value of money were also presented within the survey to the respondents (i.e., Australian female consumers). The percentages of correct responses were 71%, 55%, 50%, 43% and 62% for questions related to basic numeracy, interest rates, inflation, time value of money and money illusion, respectively. While a large proportion of respondents had some knowledge in each domain of financial literacy, only 17.64% correctly answered all of the questions. As a result, while respondents appeared to have some knowledge of specific elements of financial literacy, their overall knowledge of financial literacy concepts was very low.

### 4.2. Reliability and Validity Analysis

**Figure 3:**
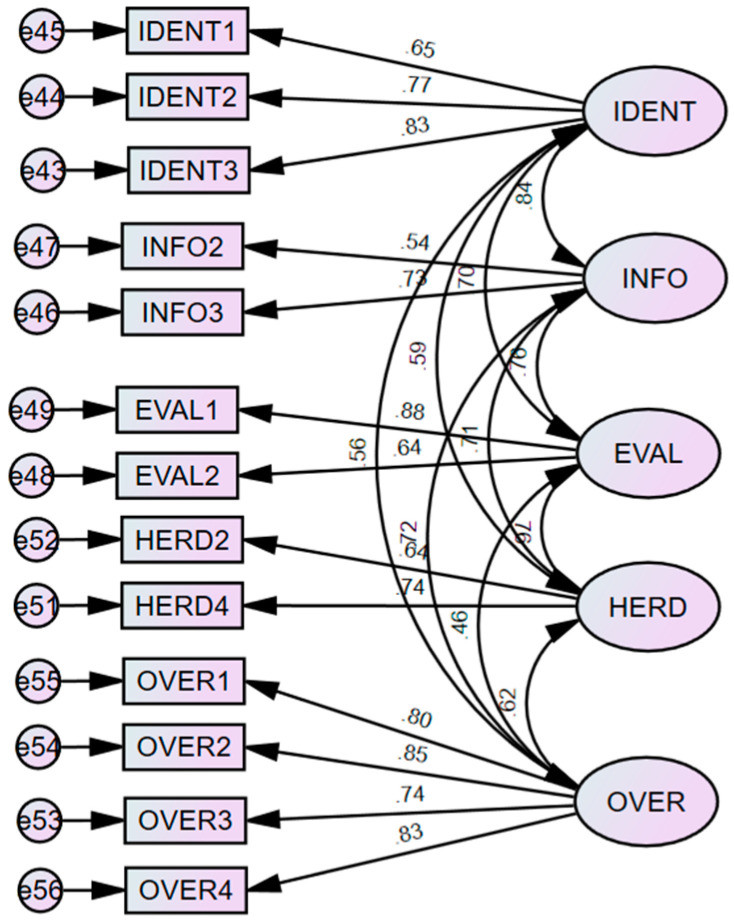
Confirmatory factor analysis.

Before conducting the primary analysis and testing of the measurement model, preliminary analyses such as tests for normality and multicollinearity were also undertaken in this study. Further, it is ensured that skewness and kurtosis values are within the acceptable range of ±1.5, which showcases that the data utilised within the study were normally distributed. Moreover, tests for common method bias were undertaken to confirm the validity of the dataset. Common method bias is “variance attributable to the measurement method rather than to the constructs the measures represent” (Podsakoff et al., 2003, p. 879). Several researchers argue that the validity of the relationships between the various constructs can only be trusted when there is no such existence of common method bias (Podsakoff et al., 2003). Further, common method bias can lead to misleading/unreliable interpretations. As a result, Harman’s single-factor tests are conducted to address the issue of common method bias (Aguirre-Urreta & Hu, 2019). In this process, exploratory factor analysis (EFA) is conducted with an unrotated factor solution to determine the amount of variance shared by the first factor. Further, Harman’s single-factor tests were performed in the current dataset, and no such common method bias was found. Later, to assess the construct’s reliability and validity, confirmatory factor analysis (CFA) was performed with AMOS 26 (See Figure 3).

Overall, results from CFA showcase that the recommended considerations have been met (chi-square = 160.483, χ2/df = 2.918, CFI = 0.94, AGFI = 0.89, RMR = 0.07, SRMR = 0.049 and RMSEA = 0.073). Several scholars suggest that the quality of each construct should be checked through both convergent and discriminant validity (Hair et al., 2010; Hubley & Zumbo, 2011; Perry et al., 2015). Convergent validity states that all the items should contribute with a higher factor loading toward the construct so that it can reflect a higher proportion of variance (Hair et al., 1998). As a result, it is confirmed that all of the observed variables had standardised loading factors of 0.40 or more (Hair et al., 2010). Moreover, studies also suggest that AVE scores that have an extraction of 0.40 or higher are acceptable when their composite reliability scores, along with Cronbach’s alpha score, are 0.60 or higher (M. C. Han, 2021; Lam, 2012; Lei & So, 2021). Based on these arguments, it is reasonable to state that all the chosen constructs in the study had good validity and reliability measures (see Table 3).

**Figure 4:**
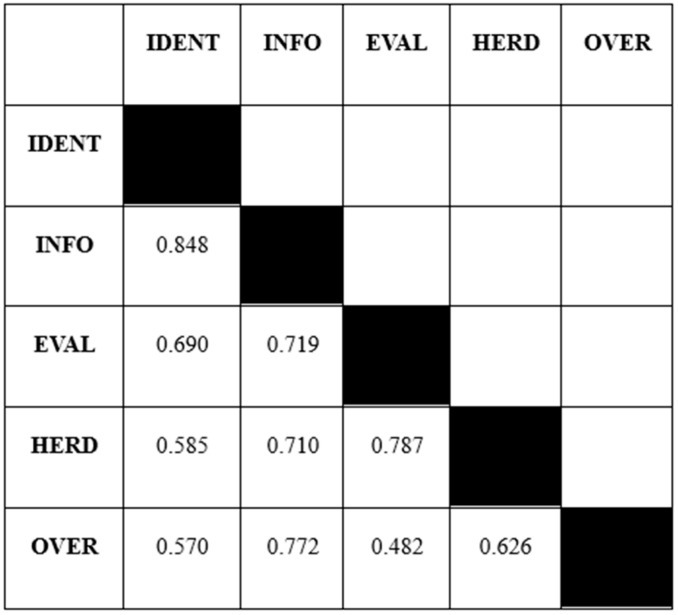
Discriminant validity.

With the increasing popularity of variance-based structural equation modelling, it is very important for researchers to test convergent validity and discriminant validity issues in a dataset (Hair et al., 1998; Henseler et al., 2015). Discriminant validity is established when the average variance explained by a construct is greater than its squared correlation estimate (Mohamad et al., 2015). Along these lines, Henseler et al. (2015) introduced a novel approach to the heterotrait-monotrait ratio of correlations (HTMT) to measure the discriminant validity within a dataset. Additionally, it is argued that a threshold of 0.85 in HTMT tests is much more reliable in stating that latent variables are different from each other (Henseler et al., 2015; Voorhees et al., 2016). As a result, results from HTMT tests confirm that all the threshold values are lower than 0.85, meaning that all the variables chosen in the study are distinct (see Figure 4).

### 4.3. Results of Structural Equation Modelling

The results from the structural equation modelling (i.e., SEM) indicate a good model fit for the proposed model (χ2 = 162.81, χ2/df = 2.85, AGFI = 0.90, CFI = 0.94, GFI = 0.93 and RMSEA = 0.07). Even though chi-square statistics and RMSEA values are widely used for model fit specifications, several studies argue that AGFI and GFI scores of 0.90 or more represent a good fit measure for a model (Aguirre-Urreta & Hu, 2019; Mohamad et al., 2015). Hence, based on the above arguments, it is possible to conclude that the structural model presented in this study had a good model fit.

**Figure 5:**
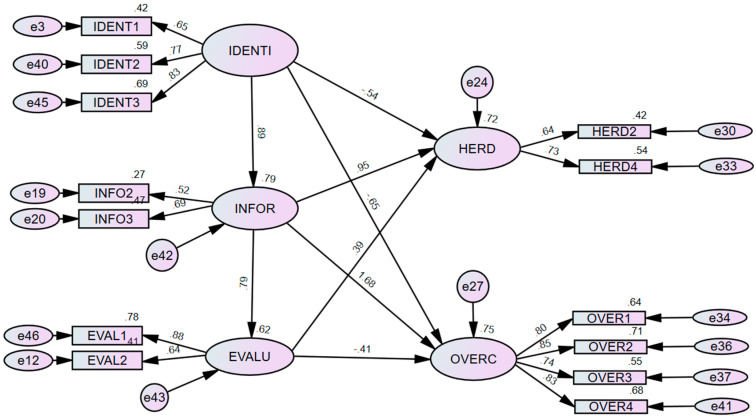
Rational decision-making and behavioural biases.

In detail, the results from structural equation modelling, as shown in Figure 5, show that all the stages involved in the rational decision-making process are statistically significant (i.e., demand identification, information search and evaluation of alternatives). Moreover, it can be inferred from the findings (i.e., H1) that an increase in one standard deviation in demand identification leads to an increase in the standard deviation of 0.89 units within the information search stages of financial decision-making for Australian female consumers. Similarly, the findings (i.e., H2) also suggest that an increase in one standard deviation in the information search leads to an increase in the standard deviation of 0.79 units within the evaluation of alternative stages of financial decision-making for Australian female consumers. As a result, the findings imply that demand identification among Australian female consumers regarding financial decision-making significantly influences the information search process, which in turn is seen to positively affect the final stage (i.e., evaluation of alternatives) of the decision-making process. Due to their frugal mentality and limited income source, it can be attributed that Australian female consumers do initially identify the financial products they want to invest in and grow their wealth. Later, Australian female consumers are believed to rely on peer members (i.e., family members/friends) or external advisors (i.e., financial advisors/investment consultants) to gather enough information before reaching a final decision. However, it can be asserted that due to behavioural biases and manipulation tactics adopted by financial advisors/consultants, Australian female consumers are influenced by biases before making their financial decisions. Hence, with consistent findings from several studies, it can be confirmed that individuals do follow the rational decision-making process (i.e., rationality) when making financial decisions (S. Kumar & Goyal, 2016; Lin, 2011; Mushinada, 2020).

More precisely, the analytical results showcase that the demand identification stage is seen to have a negative prediction of behavioural biases such as herding (β = −0.54, *p* < 0.05) and overconfidence (β = −0.65, *p* < 0.05) of Australian female consumers when making financial decisions (See Table 4). Hence, it can be argued that the demand identification stage (i.e., a need for a financial product purchase) is a purely rational approach to decision-making for Australian female consumers where their need for a financial product is not influenced by behavioural biases (i.e., herding and overconfidence). Moreover, this behaviour (i.e., frugal mentality) can be attributed to the majority of the respondents who lie within the low-income (28%) and middle-income (59%) levels and have a limited income, due to which they have a budgeting mentality and are more rational in their approach to financial decision-making (Hamilton, 2009; Servon & Kaestner, 2008).

Moreover, it can also be gathered that herding (β = 0.95, *p* < 0.05) and overconfidence (β = 1.68, *p* < 0.05) have a significant positive relationship on the information search stage of Australian female consumers when making financial decisions. In other words, it can be attributed that once the demand identification (i.e., the need for a financial product) is confirmed, Australian female consumers continue searching for information to make optimal purchase decisions. However, it can be understood that due to limited information on complex financial products and from their past experiences, Australian female consumers tend to behave irrationally. This suggests that Australian female consumers who are financially literate tend to face overconfidence bias due to their past experiences, whereas Australian female consumers who are financially illiterate tend to face herding bias when searching for information before making final product purchases (Khare & Kapoor, 2024).

Lastly, it is evident from the findings that the evaluation of alternative stages has a significant impact on herding (β = 0.39, *p* < 0.05) and overconfidence bias (β = −0.41, *p* < 0.05) when making financial decisions. In other words, this result is consistent with Lin (2011), and it can be interpreted as a view that Australian female consumers, during the evaluation of the alternatives stages, tend to be unsure (i.e., under-confidence) about their decision-making due to several reasons (e.g., lack of financial literacy, market volatilities) and are influenced by herding bias, where they tend to imitate the decisions of the other investors.

### 4.4. Role of Demographical Variables on Rational Decision-Making Process

**Figure 6:**
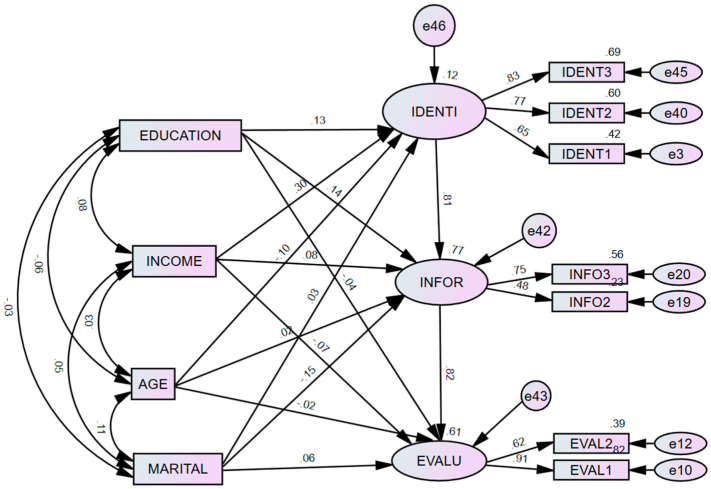
Role of demographical variables in rational decision-making process.

Within the second hypothesised model, it can be stated that demographic variables such as education, income, age, and marital status of Australian female consumers influence the rational decision-making process when making financial decisions. The results from the structural equation modelling (i.e., SEM) indicate a good model fit for the proposed model (χ2 = 102.76, χ2/df = 3.6, AGFI = 0.89, CFI = 0.92, GFI = 0.95 and RMSEA = 0.08) (See Figure 6). Further, it can be stated that education does positively influence the demand identification (β = 0.13, *p* < 0.05) and information search stages (β = 0.14, *p* < 0.05) within the rational decision-making process. Moreover, it can be argued that income (β = 0.30, *p* < 0.05) also positively influences the demand identification stages of rational decision-making. Moreover, it can be stated that the marital status of Australian female consumers (β = −0.15, *p* < 0.05) has a negative relationship with the information search stages within the rational decision-making process (see Table 5).

More precisely, results confirm that education and income significantly positively affect the demand identification stages of rational decision-making when making financial decisions. In other words, this result is consistent with previous literature, which states that Australian female consumers with higher levels of education and income levels tend to exert higher demand to invest in financial decisions so as to maximize their personal wealth (S. Kumar & Goyal, 2016). However, the analysis also reveals that age and marital status have an insignificant effect on the demand identification stages of rational decision-making when making financial decisions. Such an effect is consistent with the previous literature within the domains of financial decision-making. Additionally, results also confirm that education and marital status significantly positively affect the information-searching stages of rational decision-making when making financial decisions. In other words, this result is consistent with several literature that state that individuals with marital status (i.e., married, widowed, divorced and de facto) and who have a higher level of education tend to search for more information from friends and family members to come up with various alternatives before making a final product purchase decision. However, analysis also reveals that income and age have an insignificant effect on the information search stages of an individual’s financial decision-making. Lastly, the findings also confirm that demographic variables (i.e., age, income, education and marital status) have an insignificant effect on the evaluation of alternative stages in financial decision-making because the individuals are seen to be biased by several behavioural biases, as showcased in Figure 5 and Table 5.

## 5. Theoretical Contributions

Firstly, it can be understood that Australian female consumers are influenced by behavioural biases due to market volatility and lack of financial literacy when making financial decisions. From a theoretical standpoint, the study lays out its foundations from the adaptive market hypothesis (AMH), which conveys that rational and irrational behaviour co-exist within the market (Lo, 2005, 2019; Mushinada, 2020). Moreover, the findings provide insights into the fact that Australian female consumers are influenced by behavioural biases (e.g., herding and overconfidence) when making rational decisions due to the co-existence of financial illiteracy and intensified volatility in the financial markets. Moreover, the finding of this study is inconsistent with the propositions made by earlier theorists, such as Simon (1997), Lin (2011) and Mushinada (2020), who contend the fact that consumers tend to make satisficing financial decisions that are often rationally bounded and are driven by irrational behaviours (e.g., herding and overconfidence).

Secondly, it is argued that consumer purchase decision-making typically follows five major approaches: economic man, psychodynamic, behaviourist, cognitive and humanistic (Bray, 2008). While researchers have relied on economic, psychodynamic and behavioural approaches to provide insights into the financial decision-making process, studies have also revealed certain limitations. To address these limitations, the current study investigates consumer behaviour using a humanistic approach, which asserts that individuals do not always make decisions rationally, and their decision-making is also rationally bounded (i.e., differs from their intended decisions) (Blythe, 2013; Bray, 2008). Hence, by building upon the philosophies of Bray (2008), Lo (2005, 2019) and Mushinada (2020) on consumer decision-making, this study contributes to the understanding of how behavioural biases influence each stage of rational decision-making when making financial decisions.

## 6. Conclusions and Implications for Practice

In conclusion, it can be argued that rational actors are believed to undergo certain steps to make a financial product purchase decision. However, the current study revealed that behavioural biases (i.e., herding and overconfidence) influence the various stages involved in the rational decision-making process of Australian female consumers when making financial decisions. In doing so, the three hypothetical scenarios revealed that Australian female consumers have a conservative attitude with lower levels of financial literacy. Additionally, it can be argued that Australian female consumers are largely vulnerable segments of the population who have some knowledge of the fundamentals of financial literacy. However, their overall knowledge of financial literacy concepts is found to be very low. Hence, based on the study’s findings, it is suggested that the strategies below would allow Australian female consumers to make effective financial decisions (see Table 6).

## 7. Limitations and Future Research Directions

The findings of the study are limited to the Australian context. Given that female consumers in other countries undertake different roles and duties in their families, a comparative investigation within both genders could reveal more generalisable findings. Moreover, the current study investigated the relationships between behavioural biases (such as overconfidence and herding) and the rational behaviour of Australian female consumers when making decisions rationally. However, the role of behavioural biases, such as disposition effects and risk aversion, are excluded from this study. Hence, future studies considering these effects of behavioural biases could provide richer theoretical and practical insights. Lastly, it is suggested that future studies within this field should cover a longer period across various countries so that the decision-making powers of Australian female consumers can be understood from various cultural backgrounds and their preferences while making financial investments.

## Figures and Tables

**Table 1:** Theoretical linkages to financial decision-making among women.

Psychological Theories/Models	Key Literature	Implications in Women’s Financial Decision-Making Styles
**Theory of planned behaviour**	Sotiropoulos and d’Astous (2013)	Social class and comparison among women are observed based on excessive spending using credit cards.
**The transtheoretical model of change**	Shockey and Seiling (2004); Xiao et al. (2001)	Women tend to hold on to their investments to gain a larger profit rather than selling them, even if selling might be more profitable.
**Health-belief model**	Kaur and Vohra (2012)	Most female buyers have a sense of fear/loss when making decisions.
**Theory of reasoned action**	Ozmete and Hira (2011)	Women perceive financial decision-making as traumatic and time-consuming.
**Risk-reduction model**	Kaur and Vohra (2012); Ozmete and Hira (2011)	Women rely on financial advisors so as to minimise risk while making decisions.
**Role theory**	Hira and Mugenda (2000); Loibl and Hira (2006)	Less financial knowledge leads to a lower level of confidence in the decision-making process.

**Table 2:** Responses to three hypothetical scenarios and forecasting investment trends.

Hypothetical Scenario 1	Low Gain/No Loss	Medium Gain/Medium Loss	High Gain/High Loss
**Hypo 1:** Assume that your bank offers you an investment with the following characteristics: low gain/no loss, medium gain/medium loss and high gain/high loss. Which one would you choose?	47.33%	50.70%	1.96%
**Hypothetical Scenario 2**	Will shift to other investment products that have better performance	Will wait for some days to see any improvements in performance	Will wait for some weeks to see any improvements in performance
**Hypo 2:** Assume that you have invested some money in investment products. What do you do if their interest rates start to generate a loss?	24.64%	54.06%	21.28%
**Hypothetical Scenario 3**	Will shift to an investment with a stable return	Will wait for some days to see a stable interest rate	Will wait for some weeks to see a stable interest rate
**Hypo 3:** Assume that you have invested in some investment products. What do you do if their interest rates start to have an unexpectedly high return?	16.80%	55.46%	27.73%
**Forecasting Investment Trends**	Cannot Predict (1)		Can Predict (10)
How easy is it for you to predict the interest rates of the financial product that you have purchased recently?	80.4%		19.6%

**Note:** Your bank offers you different industry/retail funds to purchase with the following characteristics: low gain/no loss, medium gain/medium loss and high gain/high loss. Which one would you choose?

**Table 3:** Results from confirmatory factor analysis.

Construct	Items	Factor Loading	Sig	Cronbach’s (α)	CR	AVE
Demand Identification	IDENT1	0.65	***	0.79	0.79	0.57
	IDENT2	0.77	***			
	IDENT3	0.83	***			
Information Search	INFO2	0.54	***	0.60	0.60	0.41
	INFO3	0.73	***			
Evaluation of Alternatives	EVAL1	0.88	***	0.74	0.74	0.60
	EVAL2	0.64	***			
Herding	HERD2	0.64	***	0.65	0.65	0.48
	HERD4	0.74	***			
Overconfidence	OVER1	0.80	***	0.88	0.88	0.64
	OVER2	0.85	***			
	OVER3	0.74	***			
	OVER4	0.83	***			

Notes: CR—composite reliability; AVE—average variance extracted, *** *p* < 0.001.

**Table 4:** Hypothesised path relationships in the proposed model.

Hypothesis	Structural Relationships	Regression Weights	S.E.	C.R.	*p* Label	Results
***H1***	Demand Identification → Information Search	0.89	0.11	8.06	(***)	***Supported***
***H2***	Information Search → Evaluation of Alternatives	0.79	0.11	8.69	(***)	***Supported***
***H3***	Demand Identification → Overconfidence	−0.65	0.41	−2.07	(***)	***Supported***
***H4***	Demand Identification → Herding	−0.54	0.15	−2.45	(***)	***Supported***
***H5***	Information Search → Overconfidence	1.68	0.51	4.20	(***)	***Supported***
***H6***	Information Search → Herding	0.95	0.16	3.87	(***)	***Supported***
***H7***	Evaluation of Alternatives → Overconfidence	−0.41	0.17	−2.39	(***)	***Supported***
***H8***	Evaluation of Alternatives → Herding	0.39	0.75	2.90	(***)	***Supported***

Notes: * *p* < 0.05; ** *p* < 0.01, *** *p* < 0.001.

**Table 5:** Relationship of demographic variables with rational decision-making.

Structural Relationships of Demographical Variables	Regression Weights	S.E.	C.R.	*p* Label	Results
Education → Demand Identification	0.13	0.03	2.30	(***)	***Supported***
Education → Information Search	0.14	0.03	2.41	(***)	***Supported***
Education → Evaluation of Alternatives	−0.04	0.04	−0.79	0.42	***Not Supported***
Income → Demand Identification	0.30	0.03	5.0	(***)	***Supported***
Income → Information Search	0.08	0.03	1.39	0.164	***Not Supported***
Income → Evaluation of Alternatives	−0.07	0.44	−1.12	0.26	***Not Supported***
Age → Demand Identification	−0.10	0.03	−1.80	0.07	***Not Supported***
Age → Information Search	0.24	0.03	0.44	0.66	***Not Supported***
Age → Evaluation of Alternatives	−0.02	0.04	−0.32	0.75	***Not Supported***
Marital Status → Demand Identification	0.03	0.03	0.55	0.57	***Not Supported***
Marital Status → Information Search	−0.15	0.03	−2.70	(***)	***Supported***
Marital Status → Evaluation of Alternatives	0.06	0.04	1.01	0.31	***Not Supported***

Notes: *** *p* < 0.001.

**Table 6:** Summary of managerial implications.

Key Issues	Institutional/Managerial Implications
Marketing Stimuli	Managers must attempt to provide a transparent articulation of benefits associated with each financial product to entice female customers to decide rationally when purchasing their products. Financial product promotions and communication strategies should reflect higher levels of self-efficacy, allowing female consumers to believe they can achieve their desired goals.
Mechanisms	As rationality improves financial decision-making, it is suggested that the calculative benefits and returns from financial product purchases be highlighted in advertising and promotions in a way that appeals to female consumer segments. As complex financial products elicit anxiety and create uncomfortable situations, consumers are often seen to make unusual, risky decisions and gain higher decision-making powers to come out to a profitable situation. Therefore, it is strongly recommended that financial products be made easy for female consumers to understand so that they can make effective, rational decisions.
Government/Institutional	Financial literacy is still a critical challenge that needs to be addressed among both young and older females. Therefore, it is recommended that the government and other institutions deliver awareness programs focusing on developing financial literacy behaviour among Australian female consumers.

## Data Availability

The data supporting the study’s findings are available from the corresponding author upon approval from Swinburne’s Human Research Ethics Committee.
